# A Quick and Sensitive LC-MS/MS Method for Simultaneous Quantification of Sofosbuvir Metabolite (GS-331007) in Human Plasma: Application to Hepatitis C Infected Patients with End-Stage Renal Disease

**DOI:** 10.34172/mejdd.2024.375

**Published:** 2024-04-30

**Authors:** Sara Majd Jabbari, Maryam Dibaie, Khadije Maajani, Shahin Merat, Sadaf Ghajarieh Sepanlou, Mohammad-Reza Rouini

**Affiliations:** ^1^Liver and Pancreatobiliary Diseases Research Center, Digestive Diseases Research Institute, Tehran University of Medical Sciences, Tehran, Iran; ^2^Department of Pharmaceutics, Faculty of Pharmacy, Tehran University of Medical Sciences, Tehran, Iran; ^3^Department of Epidemiology and Biostatistics, School of Public Health, Tehran University of Medical Sciences, Tehran, Iran

**Keywords:** Sofosbuvir, SOF metabolites, UPLC-MS/MS, Validation, Hepatitis C, Hemodialysis

## Abstract

**Background:**

Sofosbuvir (SOF) is a revolutionary treatment for patients with hepatitis C virus (HCV). However, its efficacy and safety among patients with end-stage renal disease (ESRD) remains controversial. In this study, we examined the levels of SOF metabolite (GS-331007) (SOF-007) in human plasma of patients infected with HCV having ESRD using an optimized liquid chromatography-mass spectrometry (LC-MS) analytical method.

**Methods:**

In this case-control study, 10 clinically confirmed cases and five controls were enrolled. SOF-007 was extracted from plasma using methanol precipitation. The limit of detection (LOD) for the drug and its metabolite were 0.85 and 2.3, respectively. Such a wide range of quantification in a period of separation time shorter than 3.0 minutes (run time) allowed monitoring of the plasma concentration of analytes up to 4 hours (pre-dialysis and post-dialysis) for 12 weeks in non-cirrhotic patients with HCV infection undergoing dialysis.

**Results:**

SOF-007 in the plasma of HCV patients with healthy kidneys showed no cumulative effect. An analysis comparing patients with ESRD and healthy participants showed that their behaviour was similar, followed by dialysis with a relatively small cumulative effect.

**Conclusion:**

The plasma concentrations of SOF-007 decreased significantly after the 4-hour period of dialysis compared with the plasma concentrations hemodialysis of pre-dialysis in HCV patients with ESRD.

## Introduction

 Hepatitis C virus (HCV), the most common liver disease in hemodialysis patients, accounts for 170 to 200 million people worldwide.^1^ This chronic liver disease led by hepatitis C is among the most critical causes of mortality in kidney transplant patients (8%-28%).^2-5^ Given the high rate of disease infection among hemodialysis patients, controlling the transmission of infection and maintaining the desired quality of life in these patients is of great priority. According to some epidemiological studies, 55% to 85% of people with acute HCV infection were within the age range of 20 to 25 years, 5%-20% of them developed cirrhosis, and 30% of patients withHCV-induced cirrhosis entered the end-stage liver failure within 10 years. In 15% to 45% of cases, acute HCV infection is resolved spontaneously. Usually two decades after the inception of the persistent HCV infection, cirrhosis occurs with a high-fat diet, especially in elderly men, those who drink more than 50 g of alcohol per day, and people with obesity. In kidney transplant patients, the prevalence of hepatitis C was reported from 2.6% to 6% in different partsof the world.^1^

 Treatment of hepatitis C in patients with severe renal impairment has always been considered a challenge. Prior to the introduction of all-oral regimens, interferon-alpha, either standard or PEGylated, was the only available option for these patients. Since ribavirin is not frequently tolerated in patients with renal failure, just interferon has been prescribed despite its low response rate. Moreover, the adverse events of interferon have tended to be more severe in patients already suffering from symptoms related to renal failure and hemodialysis.^6,7^ Subsequently, advanced liver disease is known as one of the significant causes of death in patients with severe renal impairment.^2^ HCV infection has been associated with increased rates of morbidity and mortality. Although the treatments can improve the survival rate in these patients, hepatitis C can be a cause of renal disease.^3^

 Generally, direct-acting antiviral agents indicate that the progress of HCV infection treatment leads to particularly high sustained virological response rates in HCV patients.^4^ Althoughsome antiviral drugs exist for treating hepatitis C, all of them have toxic metabolism that leads to kidney and liver failures. These consequences are vital for determining the optimum level of antiviral drugs in the blood plasma but are more problematic for patients with chronic liver diseases. In this vein, sofosbuvir (SOF) is one of the most influential drugs for hepatitis C therapy.^5^

**Figure 1:**
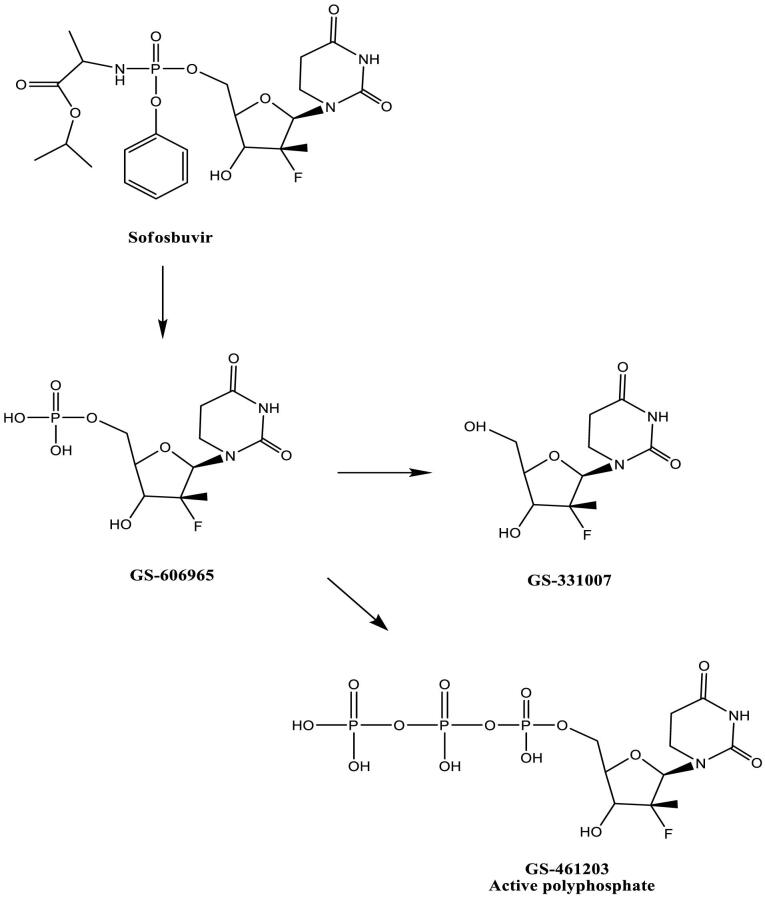
Sofosbuvir metabolic activation pathway

 SOF (Figure 1) is a phosphoramidate prodrug) β-d-2-deoxy-2ă-fluoro-2-β-C-methyluridine nucleotide) for the treatment of HCV with enhanced antiviral potency compared with earlier nucleoside analogs.^8^ It also shares some properties with the intracellular nucleoside substrates of the target HCV enzymes involved in the transcription of the viral genome. Later, when SOF is phosphorylated to the nucleoside-triphosphate, the growing HCV RNA chain is terminated prematurely during the viral replication.^6^ Compared with previous treatments, SOF-based regimens provide a higher cure rate, fewer side effects, and shorter duration of therapy.^7,9,10^

 To the best of our knowledge, no published method is available for the simultaneous quantification of SOF and its toxic metabolite (SOF-007) in biological matrices. So, the present study aimed to investigate the development of a simple, rapid, and reproducible analysis method to estimate SOF-007 in human plasma. This method is expected to provide high accuracy, sensitivity, and specificity rates by applying simple liquid-liquid extraction using ultra-performance liquid chromatography and detection via electrospray tandem mass spectrometry (UPLC-MS/MS). In order to provide a thorough analysis, which is required for a pharmacokinetic and bioequivalence study, this method should involve a very short analysis time.

 Furthermore, this method is expected to estimate the concentrations of SOF-007 in plasma samples collected from healthy volunteers after the administration of a single dose of SOF as a 400 mg pill.^11^

## Materials and Methods

###  Chemical and Reagents

 To conduct the study, SOF-007 pure standard with batch number SF.1-010218 was purchased from Khorshid Kadus Pharmaceutical Co., Iran.

 Acetonitrile and methanol HPLC grade were also purchased from Sigma Co. Other chemicals and solvents were analytical grade, while blank plasma was provided from healthy volunteers and stored at -80 ºC.

###  Patient Selection and Blood Sampling 

 In order to determine the effect of dialysis in removing the toxic metabolites of SOF, patients with hepatitis C and end-stage renal disease (ESRD) were required to refer to dialysis clinics two to three times a week based on their renal dysfunction test performed to measure dialysis adequacy. The blood samples of hepatitis patients with ESRD were collected from different dialysis centers in Tehran (Table 1). The demographic questionnaire was used to collect the participants’ information about age, sex, gender, height, weight, place of residence, socioeconomic status, treatment regimen, risk factors for HCV infection, smoking status, alcohol consumption, and family history of HCV or other viral diseases. Results of the patients’ disease history, clinical examinations, and tests performed were recorded in their medical records.

**Table 1:** Blood sampling schedule of 10 patients with chronic hepatitis C after dialysis during 12 week

**Blood Sampling table**		**Sampling of non-cirrhotic HCV patients up to 12 weeks**						**Week** **3/4/5/6**	**Week** **7/8/9/10/11**
Start the drug		The first week of dialysis after taking SOF.Cap.*			The second week of dialysis after taking SOF. Cap.			A sample per week	A sample every two weeks
A sample is taken before starting the medicine	Before dialysis	Dial. 1	Dial. 2	Dial. 3	Dial.4	Dial.5	Dial.6	A sample middle each week	A sample at the end of the ninth week and the other end of the twelfth week
		24 h	48 h	72h	96 h	120 h	144 h		
	After dialysis	28 h	52 h	76 h	100 h	124 h	148 h	A sample after 4h dialysis	A sample after 4h dialysis

*SOF. Cap.: Sofosbuvir Capsule containing 400 mg Sofosbuvir and 60 mg daclatasvir.

 The participants included 10 patients with chronic hepatitis C diagnosed by serological tests and hepatic biopsy due to chronic renal failure (eGFR ≤ 30 mL/min). These patients were selected among the individuals who were referred to the hepatitis clinic of Shariati Hospital (Table 1). Based on the treatment protocol, patients with hepatitis C were treated by Sovodak Cap. for 24 and 12 weeks if they had or had no signs of liver cirrhosis, respectively. Patients were constantly monitored and followed up on, and the necessary tests were performed throughout the treatment. The pre-dialysis and post-dialysis samples were collected during the same treatment session. As a result, 5 mL blood samples were obtained from the patients at the start of dialysis and 4 hours after. Later, the samples were transported to the hospital according to the cold chain process. After separating plasma from the blood samples, they were stored at -80 ºC. For more detailed analyses, blood samples taken from five patients with healthy kidneys were considered the control group. To prevent blood clotting, all patients were provided falcons containing EDTA and special ice packs in standard packages.

 Quantitative analysis was conducted on a *SCIEX* high-performance *liquid chromatography* (MA, USA) equipped with electrospray ionization and operated in positive ionization mode. Chromatographic separation of analytes was carried out on a Hypurity C18 column (30 × 4.6 mm, 3μm) using methanol and 2 mM ammonium Acetate buffer (pH = 3; Isocratic 90%, 10%) as a mobile phase at a flow rate of 0.3 mL.min^-1^. The column was maintained at 35ºC, and the system pressure was 6500 psi. The source-dependent parameters were maintained for the analytes and metabolites: cone gas flow, 50 L/h; desolvation gas flow, 450 L/h; capillary voltage, 5.5 kV, source temperature, 120 ºC; and desolvation temperature, 400 ºC. The optimum values of collision energy were set at 59 kV and 15 kV for SOF and SOF-007, respectively. The ions were detected in the multiple-reaction monitoring mode by monitoring the transition pairs (precursor to production) of m/z 530.21 to m/z 167.1 for SOF and m/z 261.13 to m/z 113.1 for SOF-007.

###  Preparation of Calibrators and Quality Control Samples

 Primary stock solutions (1 mg.mL^-1^) of SOF and SOF-007 were provided from separate stock solutions for the preparation of standard and quality control samples. All the primary stock solutions were prepared in acetonitrile and stored at −20 ºC for one month or more. Appropriate dilutions of the primary stock solutions were made using acetonitrile: water (50:50) at the concentrations of 2.5, 5, 10, 12.5, 15, 17.5, 20, 22.5 and 25 µg.mL^-1^ to produce stock solutions for SOF-007 and these stocks were used to prepare the calibration and quality control samples. Moreover, the calibration curve range of SOF was obtained through dilution of stock solutions at 7.8, 15.6, 31.3, 62.5, 125, 250, and 500 µg mL^-1^. Plasma standard samples were prepared by diluting the working stock solutions with blank plasma.

###  Sample Preparation

 To prepare the study samples, 500 μL of methanol was added to 200 μL of plasma samples for protein precipitation, vortexed for 1 minute, and centrifuged at 4000 g for 30 seconds. Finally, 5μL of supernatant was analyzed by a validated HPLC method.

###  Precision and Accuracy

 To increase precision analysis, three replicates at 12.5, 15, and 20 µg.mL^-1^ concentration of SOF-007 and 31.3, 62.5, and 125 µg.mL^-1^ concentration of SOF were performed on different days.

###  Eligibility Criteria and Pharmacokinetic Studies 

 Through 14 months, all patients with hepatitis C referring to the health centers were enrolled in the study if their eGFRs were under 30 mL/min/1.73 m^2^ or on regular hemodialysis. Exclusion criteria included having hepatocellular carcinoma, current or recent use of amiodarone (last 6 months), low heart rate ( < 50/min), an age lower than 12 years, previous failure of NS5A inhibitor-containing regimens, or Child-Pugh score higher than 12. Furthermore, participants without consent to take part in the study or those with mental disorders incapable of cooperating in the study were excluded. In the presence of a relative, non-cirrhotic patients were explained that the administered medicine was off-label and ensured that the research team members were closely observing and following them for the adverse events. The intervention included daily intake of Sovodak pill (400 mg SOF and 60 mg daclatasvir) at bedtime (Sovodak, Rojan Pharma co., Tehran, Iran) for 12 and 24 weeks among patients without and with cirrhosis, respectively. Cirrhosis was diagnosed by a liver stiffness > 12.5 KPa (Fibroscan, EchoSense, France) or a representation of clinical and paraclinical features, such as the presence of ascites. Moreover, splenomegaly patients with cirrhosis were treated via the same mixture for 24 weeks. Patients who had previously received anti-HCV treatment (4 weeks or more) were treatment-experienced and treated similarly to patients infected by all genotypes of HCV. The participants were followed up every other day in the first week; later, blood samples of the HCV patients were collected before and after the dialysis process. In this vein, participants were followed up weekly for up to four weeks and every four weeks thereafter. Patients were classified as lost-to-follow-up if they missed the scheduled appointments despite all the attempts made to contact them within three months, and their blood samples were consequently excluded from the study. In the case that the research team could contact the patient successfully, the causes of treatment discontinuation were recorded. In each appointment, participants were asked about any possible adverse consequences and side effects caused by the medications. They were also required to perform a simple physical exam. Side effects were considered severe if they had led to treatment discontinuation, moderate if the treatment was not discontinued but required changes in daily activities or additional treatments, and mild if no changes were required in their daily activities. The data for each follow-up was entered into a web-based system for analysis. The data collection procedure was regularly monitored by a central committee, and feedback was provided in case of protocol deviations or possible adverse events.^12,13^

## Results and Discussion

 Patients with severe renal impairment usually have slower non-renal clearance of drugs and a prolonged half-life compared with patients with normal renal function. As a result, SOF (the main component of HCV treatment regimens) was not recommended in severe renal impairment. Although SOF has low renal clearance and plasma levels are not significantly affected in renal impairment, the predominant metabolite of SOF (i.e. SOF-007) is highly excreted and increases the plasma levels up to 20 times in patients with severe renal impairment.^14^

**Figure 2:**
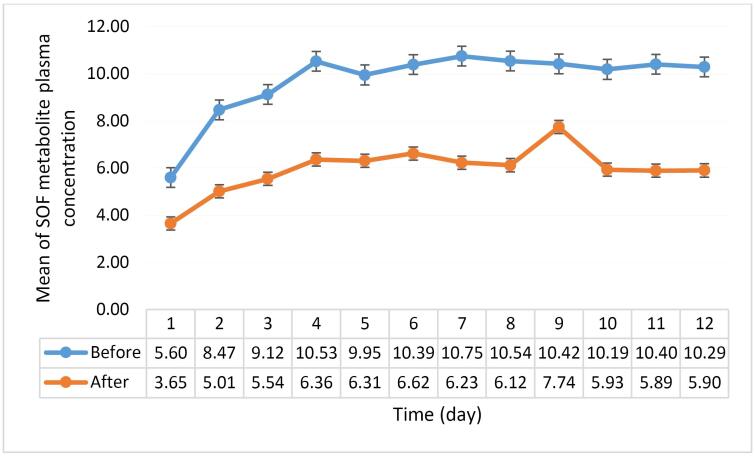
The mean accumulation diagram of SOF-007 in the plasma of 10 HCV patients’ pre- and post-dialysis

 In order to control the high plasma levels, the drug level of SOF dose (400 mg daily) was monitored. Plasma concentration-time profiles of metabolites in each patient pre-and post-dialysis showed variations in plasma metabolite concentrations during repeated oral administration of SOF (Figure 2)

 According to these profiles, the plasma concentrations of the metabolite decreased significantly after dialysis compared with pre-dialysis. The graphs of the plasma concentration ratio in post-dialysis at each sampling time also represent a significant uptake of the metabolite and a moderate steady trend after 12 days of drug administration through the dialysis procedure. These results led to a general recommendation against using SOF in severe renal impairment.

 Despite the fact that no important adverse events had been reported, the high plasma levels of SOF-007 and the lack of clinical data on its safety at the time of FDA registration led to the general recommendation against using SOF in severe renal impairments.^15^

 The unique plasma concentration of SOF-007 vs. time dominance area ratio (AUCt) in pre- and post-dialysis was calculated as 1.9 ± 0.28 (mean ± SD), indicating that dialysis could decrease the systemic exposure and toxic effects of metabolites remarkably compared with the pre-dialysis condition (*P* < 0.05).

###  Linearity and Limitation of Quantification

 A spiked solution of SOF-007 and SOF was prepared within the concentration range of 2.5- 25 µg.mL^-1^ and 7.8-500,respectively. The results indicated wide linear dynamic ranges (LDRs) for both analyses based on three replicates at each concentration level (Table 2). The limits of detection (LODs) were calculated (based on 3 S_b_/m formula) as 0.85 and 2.3 µg.mL^-1^ for SOF-007 and SOF, respectively. The precision (RSD%) of the SOF-007 determination was calculated as 6.2, 3.4, and 4.5 during the day, 7.1, 8.7, and 6.1 between days, and 12.5, 15, and 20 µg. mL^-1^ in three replications of the spiked solutions, respectively. The precision for SOF was also obtained as 10.2, 13.1, and 9.9 during the day and 9.8, 13.5, and 13.9 between days for the concentrations of 31.3, 62.5 and 125 µg.mL^-1^, respectively. The accuracy of the method was measured by adding three concentrations of SOF-007 (12.5, 15, and 20 µg.mL^-1^) and SOF (31.3, 62.5, and 125 µg.mL^-1^) into a plasma sample. The results indicated relative recoveries within the ranges of 90.7%-104.2% for SOF-007 (Table 3) and 85.7%-111.4% for SOF (Table 4).

**Table 2:** The wide linear dynamic ranges for both analytes

**Analytes LDR (µg.mL**^-1^**)**	**Equations of calibration curves**	
SOF-007	2.5- 25	Y = 5191.314 X-4935.57
SOF	7.8- 500	Y = 85.33929 X + 284.3751

**Table 3:** Daily and inter-daily changes for standard metabolite samples

**Plasma Conc. (μg.mL**^-1^**)**	**Within-Day**		**Between-Day**	
	**RSD **	**Accuracy**	**R.S.D**	**Accuracy**
12.5	6.2	97.1	7.1	90.7
15	3.4	103.3	8.7	90.8
20	4.5	104.2	6.1	98.7

RSD, Relative Standard Deviation

**Table 4:** Daily and inter-daily changes for standard SOF samples

**Plasma Conc. (μg.mL**^-1^**)**	**Within-Day**		**Between-Day**	
	**R.S.D**	**Accuracy**	**R.S.D**	**Accuracy**
12.5	31.3	10.2	111.4	108.8
15	62.5	13.1	110.0	93.0
20	125	9.9	97.6	85.7

###  Selectivity 

**Figure 3:**
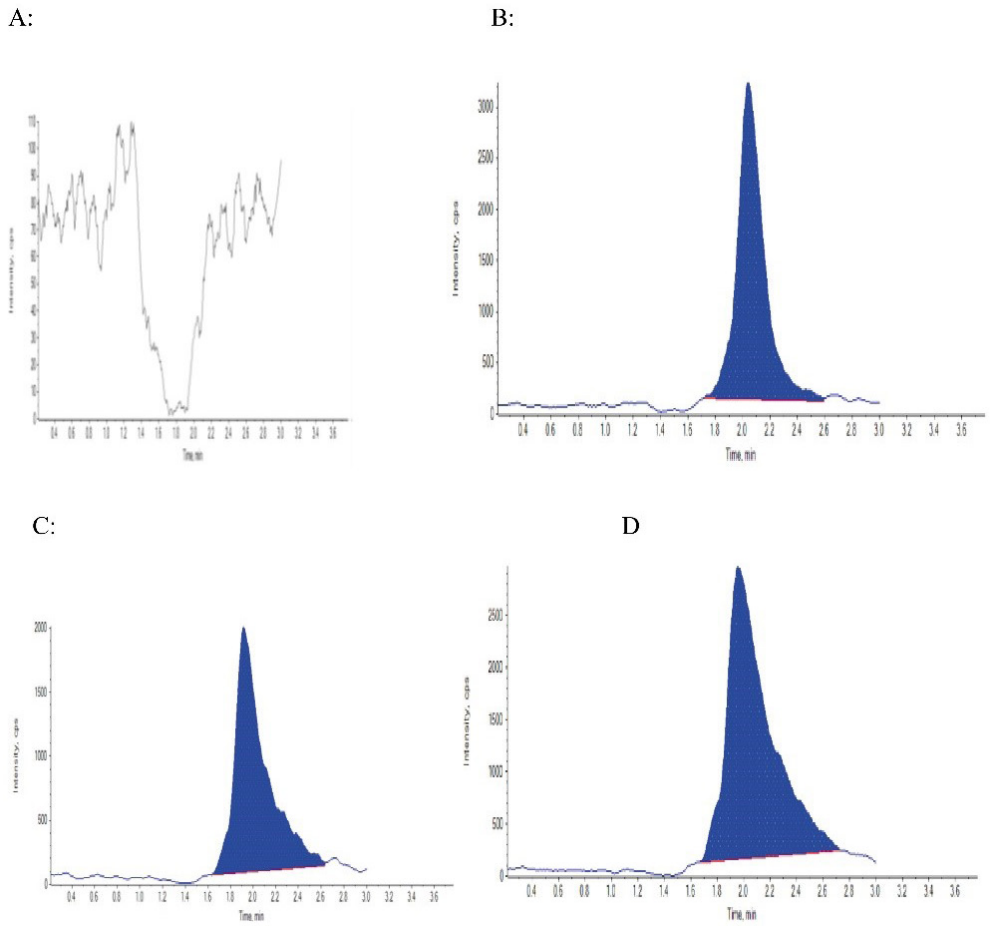
Chromatograms of (A) Chromatograms of drug-free human plasma (C = 0); (B) Drug-free human plasma (C = 10 µg/mL) / Blank plasma spiked with IS (C = 10 µg/mL); (C) Plasma sample from a subject administration 1 day of one tablet containing 400 mg SOF-pre-dialysis (time: 1 days, concentration = 8.46 μg/mL) (D) Plasma sample from a subject administration 15 days of one tablet containing 400 mg SOF-Post-dialysis (Time: 15 days, Concentration = 6.35 μg/mL )

 The selectivity of the proposed method was demonstrated by its capability to differentiate and quantify the analytes from endogenous components in the plasma matrix. Figure 3 illustrates the chromatograms of (A) drug-free human plasma (C = 0); the chromatograms of (B) drug-free human plasma (concentration = 10 µg/ml); the chromatograms of (C) plasma sample from a patient taking one tablet containing 400 mg in pre-dialysis (time: 1 day, concentration = 8.46 μg/mL), and the chromatograms of (D) plasma sample from a patient taking one tablet containing 400 mg 15 days after dialysis (time: 15 days, concentration = 6.35 μg/mL).

###  Application to Biological Samples

 The proposed analysis method was employed to determine SOF-007 in plasma samples of 10 hepatitis C patients and five healthy adult volunteers. The participants’ mean age and weight were 47 years (ranging 35-60) and 71 kg (ranging 55-85). These patients underwent a 12-week treatment, taking a Sovodak pill per day.

 Blood samples were collected from 15 volunteers, and symptoms such as fatigue, headache, and nausea were reported as adverse side effects (6%), but the results were not significant. Scholars have mentioned that SOF was extensively metabolized in the liver to form the pharmacologically active nucleoside analog triphosphate.^16^

## Conclusion

 A highly sensitive and high-quality liquid chromatography was administered to determine SOF-007 in human serum. The chromatography device was validated using the electrospray tandem mass spectrometry method. According to the findings, this method was appropriate in pharmacokinetic studies due to its sensitivity in measuring SOF-007 concentration up to 24h after dialysis and providing reliable exposure and accumulation estimates in single-dose bioequivalence studies in HCV patients with ESRD.

 To investigate concentrations of the main metabolite (SOF-007) in different days (Based on blood sampling schedule-Table S1) in each patient, pre- and post-dialysis reports were analyzed by LC/Ms. As represented in Table S1, the plasma concentration of SOF-007 is very high, and the accumulation effect of SOF-007 is observed in the patients’ bodies at the pre-dialysis stage. After dialysis, the plasma concentration of SOF-007 decreased rapidly, reaching approximately half of its initial value. Examination of patients with healthy kidneys (Table S2) showed no cumulative effect of SOF-007. A comparison between the results of Table S1 and Table S2 through Figure 2 shows that HCV patients with chronic kidney disease behave like patients with healthy kidneys followed by dialysis with a relatively small cumulative effect.

 In conclusion, the proposed method performed effectively in decreasing the poisonous concentration of SOF and its metabolites in the plasma of hepatitis C patients with severe renal impairment. The metabolite half-life was 35 hours, which is the time required to reduce metabolite concentration to half of its initial value. Compared with pre-dialysis, dialysis could reduce the plasma concentration of toxic metabolite SOF-007 by half within 4 h during the routine dialysis process.

## Supplementary Files

Supplementary file 1 contains Tables S1 and S2.
