# Supplementary material for: A Quick and Sensitive LC-MS/MS Method for Simultaneous Quantification of Sofosbuvir Metabolite (GS-331007) in Human Plasma: Application to Hepatitis C Infected Patients with End-Stage Renal Disease
Source: Middle East J Dig Dis. 2024 Apr 30;16(2):96–101. doi: 10.34172/mejdd.2024.375 (PMC11316195; doi:10.34172/mejdd.2024.375)
Supplement: Supplementary file 1 — contains Tables S1 and S2. [file mejdd-16-96-s001.pdf]

Middle East J Dig Dis. April 2024;16(2):96-S1.

10.34172/mejdd.2024.375

Supplementary file 1

Table S1: SOF metabolite plasma concentration- time in HCV patients with ESRD

Time (day)	A		B		C		D		E		F		G		H		K		I	
	before	after	before	after	before	after	before	after	before	after	before	after	before	after	before	after	before	after	before	after
1	5.66	2.95	8.46	4.01	2.24	2.39	5.75	3.6	5.78	3.57	6.01	3.24	N.D.	N.D.	4.37	5.65	7.26	4.6	4.83	2.85
3	11.5	6.07	9.94	4.34	4.98	4.68	7.2	4.8	6.73	3.89	7.6	3.88	N.D.	N.D.	8.68	7.01	12.5	6.25	7.11	4.23
5	11.5	6.35	11.9	4.73	7.16	5.6	8.1	5.62	7.02	4.33	10.4	5.51	4.22	2.49	9.83	7.23	12.8	7.2	8.27	6.34
8	11.86	7.01	10.1	5.07	9.22	8.13	9.83	6.23	6.96	2.38	12.05	6.37	5.09	3.29	12	8.54	16.3	7.63	11.89	8.96
12	N.D.	N.D.	9.03	5.09	8.95	7.3	12.2	7.05	6.31	2.43	10.5	5.29	7.24	4.9	10.1	7.86	14	8.66	11.2	8.24
15	N.D.	N.D.	8.1	5.94	10.3	5.93	12.7	9.71	5.91	2.59	10.81	5.93	8.11	5.51	12.25	7.75	13.8	7.21	11.65	9.01
19	N.D.	N.D.	7.09	5.32	10.9	6.27	11.7	6.94	6.51	2.61	12.22	6.17	9.76	5.41	13.3	7.71	13.21	7.28	12.08	8.37
26	N.D.	N.D.	7.12	4.17	11.4	6.99	11.6	7.02	6.73	2.5	14.06	6.48	7.99	4.31	12.5	8.44	11.9	7.32	11.54	7.89
33	N.D.	N.D.	7.01	4.1	10.9	7.19	11.63	7.44	7.08	2.28	13.7	6.46	6.11	3.68	14.5	7.65	12.4	6.32	N.D.	N.D.
40	N.D.	N.D.	7.06	4.06	10.55	7.29	11.57	7.37	7.51	2.6	13.03	7.08	6.26	3.71	12.44	6.44	12.24	6.48	11.01	8.37
48	N.D.	N.D.	7.02	4.09	10.61	6.81	11.60	7.45	6.39	2.56	13.15	6.95	6.18	3.57	14.8	6.53	12.52	6.24	11.34	8.77
62	N.D.	N.D.	7.07	4.07	10.60	6.72	11.65	7.59	6.03	2.63	13.09	6.83	6.08	3.64	13.78	6.62	12.33	6.38	11.95	9.09

Table S2. SOF metabolite plasma concentration in HCV patients with health kidney

Time (day)	L	M	N	O	P
1	1.94	2.68	3.46	2.23	2.63
3	2.31	2.62	2.58	-	-
5	-	-	-	2.86	3.46
8	2.8	2.72	2.63	2.78	2.73
12	2.37	2.88	2.93	2.38	2.8
15	-	2.74	-	2.63	2.98
19	2.26	-	2.61	-	-
